# Patient Satisfaction With Dental Implants in the Upper and Lower Arches Placed in a Tertiary Care Setting: A Qualitative Study

**DOI:** 10.7759/cureus.96471

**Published:** 2025-11-10

**Authors:** K Shreya, Shankar S Menon, Reshma Suresh, Vineetha Karuveettil, K V Arun, Biju Balakrishnan, Maya Rajan Peter

**Affiliations:** 1 Periodontics, Amrita School of Dentistry, Amrita Vishwa Vidyapeetham, Kochi, IND; 2 Public Health Dentistry, Amrita School of Dentistry, Amrita Vishwa Vidyapeetham, Kochi, IND

**Keywords:** aesthetic outcomes, dental implants, overall treatment experience, patient satisfaction, qualitative research

## Abstract

Background: Dental implants offer functional and aesthetic rehabilitation for edentulous patients. Despite high survival rates, patient satisfaction hinges on more than osseointegration of titanium. This study explores subjective patient satisfaction with implants in maxillary and mandibular anterior and posterior regions in a tertiary care hospital, emphasizing aesthetic, emotional, and logistical dimensions.

Methods: A qualitative design was used involving in-depth semi-structured interviews with 30 patients (17 females, 13 males; aged 35-80) who had received dental implants at least six months prior. Transcripts were thematically analysed using QDA (Qualitative Data Analysis) Miner Lite software (Provalis Research, Montreal, Canada). Coding was based on five predefined domains: overall treatment experience, treatment coordination, perceived aesthetic outcomes, functional challenges, and review and recommendations.

Results: Of 30 patients, 26 reported an overall positive experience, one was neutral, and three were dissatisfied. Dissatisfaction arose from delays, travel difficulties, language barriers, and aesthetic mismatches. Emotional responses were positive: patients expressed joy in regaining chewing ability, confidence from restored appearance, and gratitude for empathetic care. Conversely, disappointed patients with aesthetic mismatches said they were not likely to recommend the centre or the treatment.

Conclusion: Implant success must be evaluated holistically, incorporating patient emotions, expectations, and experience. Understanding the full patient journey, including non-clinical barriers, can help practitioners refine care delivery, communication, and prosthetic planning.

## Introduction

Dental implants are widely recognized as the gold standard in the rehabilitation of partial and complete edentulism, with consistently high survival rates ranging from 90% to 98% over a period of 10 years [1]. These remarkable success rates are largely attributed to advances in biomaterials, surgical protocols, and prosthetic technologies that ensure predictable osseointegration and biomechanical stability [2]. Patient satisfaction has increasingly emerged as a critical metric of implant success, extending beyond the biomechanical into the psychological, functional, emotional, and aesthetic domains [3]. The dental profession, once primarily focused on structural restoration, is now compelled to account for the patient’s subjective experience of care. This evolving perspective mirrors a broader paradigm shift in healthcare that places growing emphasis on patient-centred outcomes alongside traditional biomedical indicators [4]. Discrepancies between clinical and patient aesthetic assessments can significantly influence satisfaction, especially in single-tooth anterior restorations [5]. From the clinician's perspective, objective parameters such as crown contour, colour match, or gingival zenith might be deemed acceptable. Yet, patients may judge the same results through a personal and emotional lens, anchored in their social self-image and everyday interactions. Beyond these individual perceptions, Social Determinants of Health (SDOH) - including income, education, occupation, and access to care - also shape how patients experience and evaluate dental implant treatment. In the Indian context, socioeconomic disparities, cultural norms, and health literacy levels influence not only treatment accessibility but also expectations of aesthetics and function. For instance, patients from higher socioeconomic backgrounds may prioritize esthetics and advanced materials, while those from lower-income groups often emphasize affordability and chewing comfort. Recognizing these broader social factors allows clinicians to adopt a more holistic, patient-centred approach, ensuring that implant therapy outcomes are both clinically successful and socially meaningful.

Patient satisfaction is a multidimensional construct. It encompasses a range of factors, including aesthetic appeal, chewing comfort, speech clarity, treatment experience, emotional resilience, and the nature of interpersonal interactions with the practitioner [6]. The integration of patient-reported outcome measures (PROMs) into implant dentistry reflects a commitment to understanding and responding to patients’ lived experiences [7]. Despite this theoretical shift, much of the existing literature remains heavily skewed towards quantitative metrics such as marginal bone loss, peri-implant mucosal health, implant mobility, and radiographic success [8]. While these are undeniably important, they may not always align with what matters most to the patient. Numerous studies have reported high levels of general satisfaction with implant-supported prostheses [9, 10]. These results, however, are often derived from structured questionnaires or checklist-based instruments that may not capture the emotional, contextual, and dynamic factors that shape the implant journey [11]. For example, two patients may both rate their satisfaction as “very high” but for completely different reasons-one may value aesthetic congruence with adjacent teeth, while another may prioritize restored function or relief from denture instability. Furthermore, these tools may be ill-equipped to capture subtle forms of discontent. A patient may endorse satisfaction due to social desirability bias, or because they assume that no better outcome was possible. In this sense, qualitative methodologies offer a deeper lens through which to understand the subjective implant experience [12]. Unlike quantitative surveys that restrict responses to predefined options, qualitative interviews allow patients to articulate what matters most to them in their own terms.

Aesthetics, particularly in the anterior maxillary region, are frequently cited as pivotal determinants of satisfaction. The anterior zone holds significant weight in facial aesthetics, smile dynamics, and interpersonal communication. Even minor colour mismatches, lack of incisal translucency, or subtle gingival asymmetry can be distressing for patients [13]. Patient evaluations of implant aesthetics are as critical as those of dental professionals [14]. In practice, patients with anterior implants tend to scrutinize their smile more intensely, even identifying features that the clinician may have dismissed as clinically irrelevant or impossible to modify. On the other hand, posterior implants, though less visible, play a critical role in restoring function and dietary independence. For many edentulous or partially edentulous individuals, the loss of posterior teeth compromises their ability to chew efficiently, which can lead to altered food choices, nutritional deficiencies, and even digestive issues. The placement of implants in these regions can markedly improve masticatory performance, enhancing not only physical well-being but also psychosocial health by enabling participation in social meals and removing the stigma of denture-related limitations [15, 16].

Functionality and aesthetics intersect with psychosocial outcomes in complex ways. Tooth loss is often accompanied by profound emotional consequences-embarrassment, social withdrawal, avoidance of smiling or speaking, and lowered self-esteem [17]. Implants are seen not only as prosthetic replacements but as symbols of renewal and restoration [18]. The emotional dimension of satisfaction is closely tied to expectation management and the quality of clinician-patient communication. Patients who feel adequately informed, involved in decision-making, and heard throughout the treatment process tend to report higher satisfaction levels, even in the presence of minor complications [19]. Conversely, when communication is insufficient or dismissive, dissatisfaction may arise despite excellent clinical results [20]. Language and cultural barriers present additional challenges in the Indian context. With over 22 official languages and numerous dialects in the country, it is not uncommon for patients and clinicians to lack a shared linguistic framework. Such mismatches can lead to miscommunication during consent, poor comprehension of post-operative instructions, and a reluctance to voice concerns [21]. Cultural factors also play a role: patients may avoid expressing dissatisfaction out of respect for authority figures or fear of offending the doctor. These undercurrents can create a disconnect between reported and actual satisfaction, especially when patients are evaluated through rigid survey tools. System-level factors are equally influential. These include appointment scheduling, waiting times, hospital accessibility, administrative efficiency, and the overall patient journey through the healthcare system. Patients attending tertiary care hospitals often face long travel distances, crowded outpatient departments, delayed treatment timelines, and multiple consultations-all of which contribute to physical, economic, and emotional burden [22, 23].

Standardized PROMs such as the Oral Health Impact Profile (OHIP-14), the General Oral Health Assessment Index (GOHAI), the Patient Health Questionnaire-9 (PHQ-9) Form, and the Prosthesis Evaluation Questionnaire are widely used in clinical research to evaluate outcomes [24, 25]. Qualitative studies, although fewer in number, offer a powerful counterbalance to purely quantitative research. By enabling patients to narrate their experiences in their own words, such studies uncover new themes, emotions, and contradictions that enrich our understanding of satisfaction. Patient satisfaction is not a static endpoint, but a dynamic interplay of past suffering, present recovery, and future expectations [20]. Moreover, patient satisfaction is often contextual, shaped by socioeconomic conditions, cultural beliefs, peer narratives, and previous dental trauma. It is also important to consider the financial aspect of implant therapy, which can significantly shape a patient’s perception of value and satisfaction. While many patients perceive implants as a long-term investment in oral health, others may find the cost excessive. Patients who experience complications or extended treatment durations may feel the high cost was not justified, even if clinical outcomes are acceptable in the end. Conversely, those who see a dramatic improvement in lifestyle may consider the expense worthwhile. Additionally, differences in satisfaction levels can emerge between patients undergoing single-tooth replacements and those receiving full-arch rehabilitations. Full-arch cases, though offering complete functional restoration, often involve longer timelines, more complex surgical protocols, and higher financial costs. These factors can influence overall satisfaction differently from single-tooth cases, where outcomes may be more straightforward and immediate.

In designing the present study, we sought to explore the lived experience among a demographically diverse cohort of patients treated with dental implants in both anterior and posterior regions of the maxillary and mandibular jaws. Unlike private clinics, where patient flow is more controlled and clinician continuity maintained, our study was in a tertiary centre, which is often plagued by fragmented care teams, lengthy procedural steps, and bureaucratic delays. These system-level realities have profound implications for patient experience, which is an area that remains underrepresented in the literature.

In totality, this study aims to transcend the binary of “satisfied” vs. “unsatisfied” by delving into the multiple layers that compose the implant experience. Here, we sought to assess patient satisfaction through five prior coded domains. Through such a qualitative approach, we aim to give voice to the emotional, aesthetic, functional, and logistical components that define satisfaction in real-world clinical practice. By centering the patient narrative, we not only reflect on the strengths of current implantology practices but also offer a roadmap for improvement, which is anchored in empathy, efficiency, and clear communication. The goal is not just to place a successful implant, but to deliver care that is meaningful, affirming, and transformative for the patient.

## Materials and methods

Study design

This qualitative study was designed to understand the lived experiences of patients who underwent dental implant therapy. It was conducted using an interpretivist paradigm, aiming to explore participants’ subjective experiences through thematic analysis rooted in their lived realities. The study took place in the periodontics department of Amrita School of Dentistry, Amrita Vishwa Vidyapeetham, Kochi, India, with a patient population reflecting a wide socioeconomic, linguistic, and geographic background. This study was conducted in accordance with the ethical standards of the institutional research committee vide no. EC/NEW/INST/2023/KL/0379 from December 2023 to November 2024 over a period of 12 months. Written informed consent was obtained from all participants.

Participant selection

A purposive sampling strategy was employed. Inclusion criteria included patients who had received at least one dental implant in either the anterior or posterior maxillary/mandibular regions, had completed their prosthetic restoration at least six months prior to the interview, and were willing to share their experiences in an interview setting. Exclusion criteria included patients with a history of systemic conditions like osteoporosis, patients with a history of radio/chemotherapy, patients with only posterior implants, and patients with implants only on one arch. A total of 30 patients were enrolled (17 females, 13 males), aged between 35 and 80 years. Participants represented diverse linguistic and socioeconomic backgrounds, with some traveling over 100 kilometres for treatment. 

Data collection 

The interviews were conducted by trained researchers with prior experience in qualitative interviewing. Efforts were made to minimize researcher bias by maintaining a neutral tone and encouraging open-ended responses. Double coding and peer debriefing were employed to enhance credibility. To ensure trustworthiness, dual coding was conducted independently by two researchers and compared for consistency. While member checking was not employed, the codes and themes were discussed and validated through peer debriefing sessions. Semi-structured interviews were conducted by a trained investigator using a flexible interview guide. Questions focused on: Overall treatment experience, aesthetic satisfaction, functional outcomes (chewing, biting, speech), logistical issues (appointments, travel, waiting times), communication and emotional responses, and willingness to recommend the hospital or treatment to others. Interviews lasted 20-45 minutes and were audio-recorded. Interviews were conducted in English or Malayalam. The entire interview questionnaire has been detailed in the Appendices section.

Thematic analysis

Transcripts were anonymised and analysed using QDA (Qualitative Data Analysis) Miner Lite software (Provalis Research, Montreal, Canada). Coding was performed by two researchers independently and compared for consistency. Five broad thematic categories were used: overall treatment experience (positive, neutral, negative), treatment coordination (waiting times, intervals, hospital delays), perceived aesthetic outcomes (natural appearance, colour mismatch, harmony), functional challenges (chewing, biting, food impaction), and review and recommendations (would recommend vs. would not). We used the Standards for Reporting Qualitative Research (SRQR) reporting guideline (O’Brien et al., 2014) to draft this manuscript, and the SRQR reporting checklist when editing; this is included in the appendix [26, 27].

## Results

A total of 30 patients (17 females, 13 males; aged 35-80) who had reported to the outpatient department of the tertiary care centre and had completed their dental implant treatment at least 6 months prior were selected. The participants had received at least one dental implant in either the anterior or posterior maxillary/mandibular regions and had completed their prosthetic restoration at least six months prior to the interview. Of the 30 patients interviewed, 26 expressed an overall positive experience, one reported a neutral experience, and three described their experience as negative. The narratives revealed a complex interplay of emotional, aesthetic, functional, and logistical factors shaping satisfaction. The following are detailed patient accounts organized by the five thematic categories, which capture the depth and diversity of their experiences.

Overall treatment experience

The majority of patients described their dental implant journey as transformative, which intertwined restored oral function and aesthetics with a revitalized sense of confidence. For many, the implants alleviated the emotional burden of tooth loss and liberated them from the stigma and self-consciousness that had constrained their interactions. Several patients expressed their joy in reclaiming everyday pleasures like eating freely, smiling openly, or speaking without hesitation, by describing these as milestones that restored their identity and dignity. These experiences resonated emotionally with the patients, as some of them likened their implants to a “second chance” at life, enabling them to reconnect with family and friends. However, a few articulated their dissatisfaction, which were marked by feelings of disempowerment and frustration. These negative experiences often stemmed from unmet aesthetic expectations, logistical hardships, or communication barriers, which made patients feel sidelined or unheard in their own treatment process. Despite such challenges, some demonstrated resilience by rationalizing their struggles and focusing on functional gains or clinical success.

Patient 14 (Female, 48 years, Anterior Maxillary Implant)

“I used to laugh with my hand over my mouth. After the implant, I smile confidently. I was sceptical at first. I thought, ‘How can an artificial tooth feel real?’ But the implant blends so well, I forget which tooth is artificial. It feels like a second chance at smiling heartily in life.”

Negative experiences, though less common, were marked by feelings of disempowerment and frustration, often tied to unmet expectations or communication barriers. These patients felt sidelined in their treatment journey, which amplified their dissatisfaction.

Patient 8 (Male, 55 years, Posterior Mandibular Implant)

“The implant works fine, but getting here was very difficult. I live in a village three hours away. Moreover, I don’t speak English well, and the doctor spoke in broken Malayalam to communicate with me over the phone. Because of this, I couldn’t ask questions properly - I just said yes to everything because I was confused.”

These negative narratives highlight emotional disempowerment, often stemming from aesthetic mismatches, logistical burdens, or language barriers, as supported by prior research on patient experiences [21, 22]. Despite these challenges, some patients expressed resilience, rationalizing their struggles in light of positive clinical outcomes.

Treatment coordination

Logistical challenges profoundly shaped patients’ experiences with dental implant therapy in a tertiary care setting, with delays, long waiting times, and travel burdens emerging as dominant themes, particularly for those from rural areas. Patients frequently voiced frustration over unpredictable appointment schedules, prolonged intervals between treatment stages, and unexpected cancellations, which disrupted their lives and tested their patience. For many, the physical and financial toll of traveling long distances, often involving early departures, extended hospital stays, and missed work, compounded these difficulties, creating a sense of exhaustion and alienation. The complexities of navigating a high-volume hospital environment, marked by bureaucratic inefficiencies, further intensified these struggles, leaving some patients feeling that their time and effort were undervalued. However, resilience was evident among others who rationalized these challenges as an acceptable trade-off for accessing specialized care at a reputable institution. These patients expressed trust in the hospital’s expertise, viewing delays as a necessary part of ensuring quality outcomes. This tension between logistical frustrations and steadfast commitment to treatment underscores the multifaceted nature of patient experiences in tertiary care, where systemic constraints coexist with clinical excellence.

Patient 16 (Male, 50 years, Posterior Maxillary Implant)

“The wait between the surgery and getting the crown was too huge: three months! Sometimes I waited weeks for the next step. The doctors were busy, and I could see they were trying. When I finally got the implant, it was worth it. I’d rather wait for quality than rush and regret it.”

Patient 22 (Female, 67 years, Posterior Mandibular Implant)

“I live far away, and the travel was exhausting. Once, I waited two hours just to see the dentist for five minutes. But I kept thinking, ‘This is a big hospital; they know what they’re doing.’ That trust kept me going, even when I was frustrated.”

These accounts reflect a tension between logistical inefficiencies and patient resilience, a common theme in tertiary care settings where high patient volumes and bureaucratic hurdles often disrupt care delivery [23]. Patients who rationalized delays often did so because of trust in the hospital’s expertise or satisfaction with the final outcome.

Perceived aesthetic outcomes

Aesthetic outcomes were a pivotal factor in shaping patient satisfaction with dental implants, particularly for the 19 patients with anterior maxillary or mandibular implants, where visual harmony significantly influenced self-perception and social interactions. Most patients expressed delight with the natural appearance of their implants, especially the seamless integration of colour, shape, and contour that restored their smiles to a level often surpassing their original teeth. This aesthetic success alleviated long-standing insecurities, boosting confidence and enabling expressing of joy in personal and professional settings. However, a minority reported dissatisfaction, primarily due to colour mismatches or gingival disharmony, which disrupted the anticipated aesthetic ideal. These patients described a spectrum of emotional responses, from mild self-consciousness to deep distress, with some avoiding mirrors or photographs due to perceived flaws. The emotional toll of these aesthetic shortcomings underscored their disproportionate impact on overall satisfaction. Despite such challenges, some patients maintained conditional satisfaction, valuing functional benefits while grappling with aesthetic concerns.

Patient 1 (Female, 37 years, Anterior Maxillary Implant):

“My implant looks better than my original tooth! I used to have a crooked front tooth, and I would cover my mouth when I talked. Now, it’s so natural, same colour, same shape. It’s like a dream come true.”

Patient 26 (Female, 45 years, Anterior Maxillary Implant):

“I saved up for this implant thinking it would fix my smile, but it has ruined it. The crown colour doesn’t match. I avoid mirrors now, and haven’t taken a photo in months.”

Patient 15 (Male, 58 years, Anterior Maxillary Implant)

“The tooth itself is great, but the gum around it doesn’t look right - it’s a bit uneven compared to my other teeth. It bothers me when I smile close-up. I still like the implant, but I notice that flaw every day.”

Aesthetic dissatisfaction can disproportionately affect overall satisfaction, particularly for anterior implants where visual harmony is paramount. Patients with minor aesthetic concerns often expressed conditional satisfaction, while those with significant mismatches reported profound emotional distress.

Functional challenges

Functionality emerged as a cornerstone of patient satisfaction with dental implants, particularly for those with posterior implants, where chewing efficiency and dietary freedom were paramount. Patients described their pre-implant lives as severely restrictive, marked by pain, limited food choices, and social embarrassment due to impaired mastication or speech. The restoration of these functions was transformative, enabling them to enjoy a diverse diet - from hard fruits to tough meats - without discomfort, and to speak clearly, fostering renewed confidence in social and professional settings. This regain of normalcy was often equated with emotional liberation, as patients expressed profound relief and a restored sense of dignity, no longer hindered by the fear of denture slippage or mumbled speech. However, the transition to full functionality was not seamless for all; some faced initial adaptation challenges, including discomfort, a foreign sensation, or food impaction, requiring adjustments like cautious chewing or enhanced oral hygiene routines. Despite these hurdles, patients viewed such inconveniences as minor compared to the profound benefits of dietary independence and social ease, underscoring the life-changing impact of restored oral function.

Patient 7 (Male, 49 years, Posterior Mandibular Implant):

“Before the implant, I was chewing only on my left side, and my jaw would hurt. I would avoid hard foods. The first week after surgery was strange - the implant felt heavy. I was scared to chew on it. But after a month, it was like my own tooth. Now, I can eat anything - fruits, vegetables, and even tough meat. It’s not just about food; it’s about enjoying life without worrying about my teeth.”

Patient 28 (Female, 57 years, Posterior Mandibular Implant):

“The implant is great, but I had to learn how to use it. At first, I was cautious, sticking to soft foods. Sometimes food gets trapped, which is annoying, but I’ve got a routine now with flossing. It’s a small trouble compared to how difficult it was before.”

These accounts echo the transformative impact of restored function, as noted in prior studies [16], while also highlighting the adaptation period required for some patients. Emotional relief was often tied to regaining social confidence and dietary independence.

Review and recommendations

When asked whether they would recommend implant therapy at the tertiary care hospital, 27 of the 30 patients said yes, reflecting strong overall approval despite logistical or aesthetic challenges. The dissenting patients cited aesthetic dissatisfaction as a dealbreaker. Positive recommendations were often framed as a desire to share the transformative benefits of implants with others [20, 24, 25].

Patient 6 (Male, 50 years, Posterior Maxillary Implant)

“Even with the delays, I would recommend it. The implant works perfectly now, and I can chew anything. The hospital was crowded, and I waited a lot, but the doctors did a great job.”

Patient 5 (Female, 45 years, Anterior Maxillary Implant)

“I wouldn’t tell others to come here. I expected a natural smile, but I didn’t get it. The crown looks too artificial, and it has changed how I see myself. I spent so much money and time, and I’m still not happy. I would rather warn people to check the colour first.”

Table 1 gives an analysis of the qualitative data obtained from QDA Miner Lite. It reveals that the majority of patients reported a positive overall treatment experience, with high levels of satisfaction regarding aesthetic outcomes and the natural appearance of their implants. Functional challenges were common, with a large number citing difficulty in biting and chewing, indicating ongoing adaptation issues despite overall contentment. Aesthetic concerns like colour mismatches and issues like food accumulation reflect areas for clinical improvement. Time-related concerns, such as long waiting times and extended intervals between appointments, emerged as prominent sources of dissatisfaction, especially in the tertiary care setting. Despite these logistical and functional hurdles, a vast number of patients stated they would recommend implants to others, underscoring a generally favourable perception. These suggest that satisfaction is shaped not only by clinical outcomes but also by the broader care experience.

**Table 1:** Frequency of Themes Identified Among Participants The count mentioned in the table refers to the number of times a particular response has been repeated by a participant in the same answer. For instance, "natural-looking" has been mentioned 26 times by the participants. Cases refer to the exact number of participants who spoke about a particular issue/question/code. For example, "natural-looking" has been mentioned by 23 participants. Hence, the same patient(s) is/are likely to have mentioned this code multiple times, which is why the count is 26 but the cases are 23 in number. Therefore, in several instances, one can observe the "Count" outnumbering the "Cases". A positive response is a response given by the patient which is completely in the affirmative (refer to Q2. in the 'Interview Questionnaire' section of the Appendices). Such a patient is fully satisfied with the treatment experience and has given a resounding 'Yes' to the interviewer. On the other hand, a negative response is elucidated when the patient is dejected by the treatment experience and explicitly mentions so. A neutral response is a response wherein the patient gives both good and bad aspects about his/her experience and refuses to outrightly pick a side.

Codes	Count	Codes (%)	Cases (%)	Cases (%)
Overall Treatment Experience				
Positive response	28	11.80%	26	86.70%
Negative response	3	1.30%	3	10.00%
Neutral response	1	0.40%	1	3.30%
Treatment Coordination				
Interval between appointments	21	8.90%	21	70.00%
Waiting time	27	11.40%	25	83.30%
Perceived Aesthetic Outcomes				
Aesthetic satisfaction	25	10.50%	25	83.30%
Colour differences	7	3.00%	7	23.30%
Natural-looking	26	11.00%	23	76.70%
Functional Challenges				
Difficulty in biting	29	12.20%	28	93.30%
Difficulty in chewing	25	10.50%	25	83.30%
Food accumulation	15	6.30%	15	50.00%
Review and Recommendations				
Would recommend	27	11.40%	27	90.00%
Wouldn’t recommend	3	1.30%	3	10.00%

**Figure 1:**
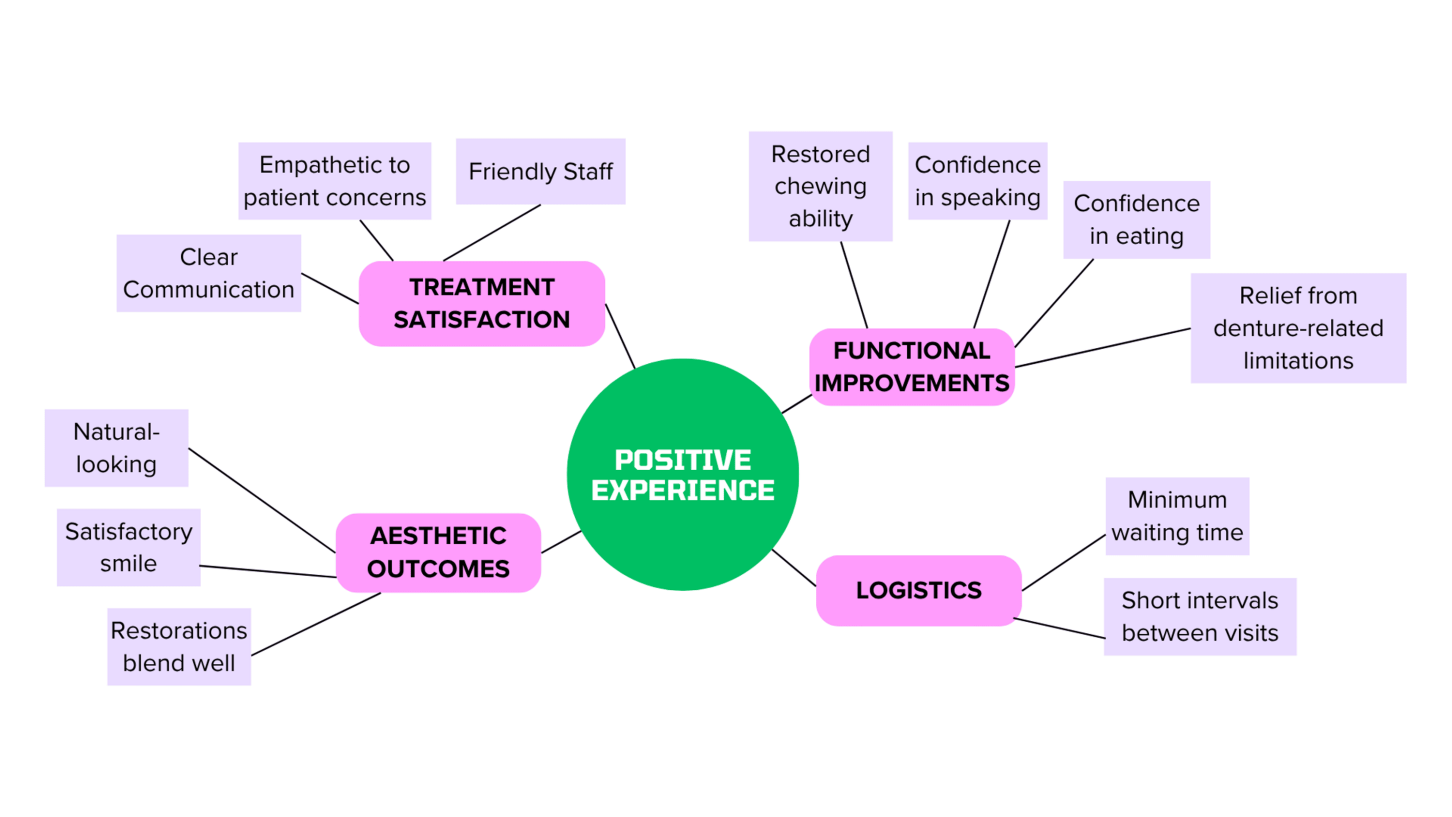
Flowchart of factors contributing to a positive patient experience Image Credits: Dr. Shankar S Menon

**Figure 2:**
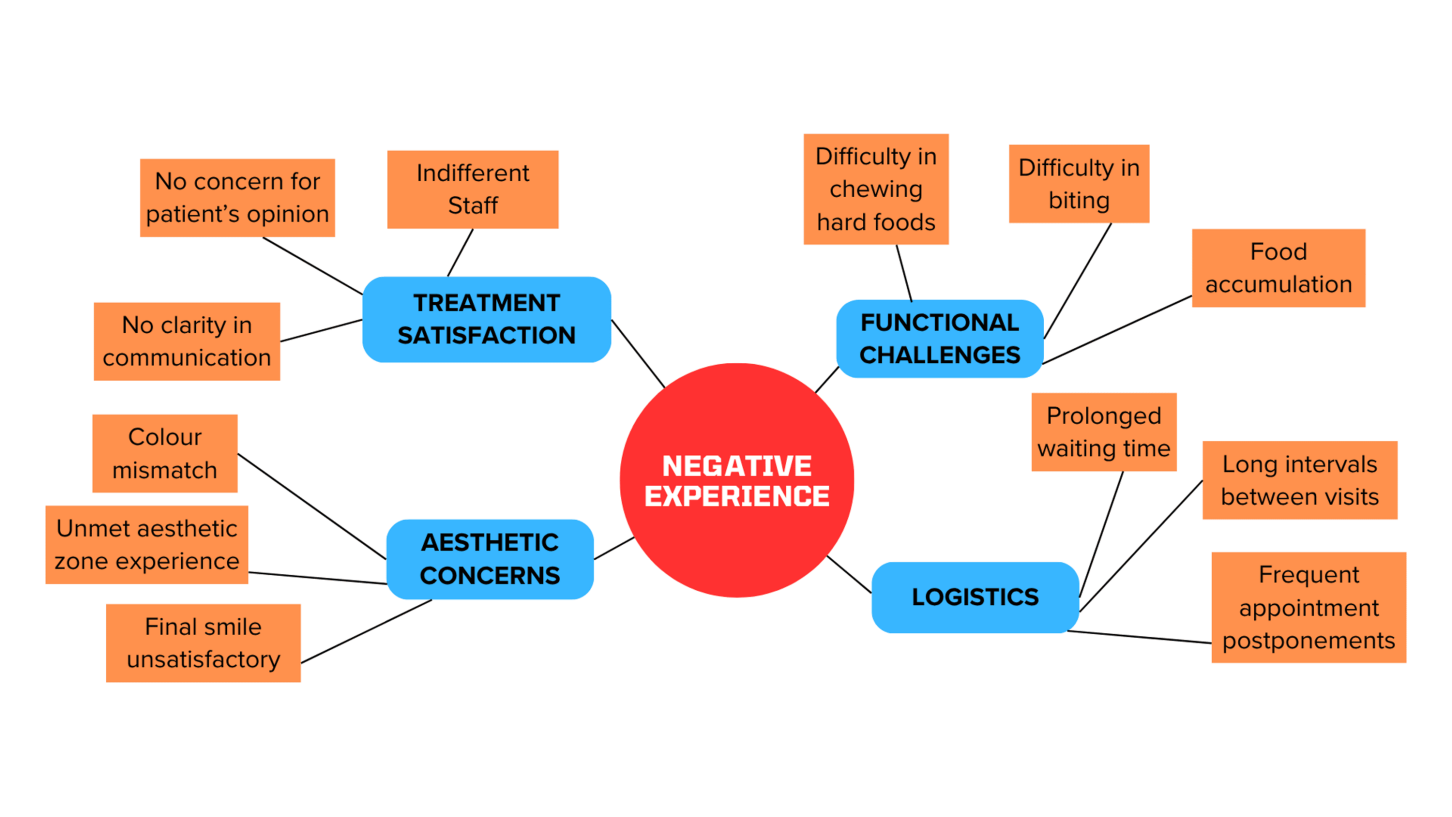
Flowchart of factors contributing to a negative patient experience Image Credits: Dr. Shankar S Menon

Figures 1-2 are flowcharts showing the various pointers for patients with regard to their experience. While Figure 1 outlines factors contributing to a positive patient experience, Figure 2 outlines those factors that contribute to a negative patient experience.

## Discussion

This study explored the multi-dimensional landscape of patient satisfaction following dental implant therapy in a tertiary care setting, extending beyond conventional clinical outcomes to encompass aesthetic, emotional, functional, and systemic experiences. Our findings underscore the necessity of redefining implant success to include patient narratives, especially in settings with structural and linguistic barriers. 

The overwhelming majority of participants (26 out of 30) reported positive satisfaction, which aligns with previous literature on the high satisfaction rates associated with implant-supported prostheses [9, 10]. However, this apparent homogeneity masks the complex emotions and contextual factors that shaped each participant’s implant journey. As observed in prior qualitative investigations [12, 20], patient satisfaction is seldom a straightforward outcome; it is a product of evolving expectations, personal history, and healthcare interactions. 

Participants’ narratives often reflected deep emotional transformations, echoing prior findings that implants can restore more than just function-they often revive identity and social participation [17, 18]. Several patients described renewed confidence in social settings, restored dignity, and a sense of normalization post-treatment. Conversely, negative experiences, though fewer, were emotionally striking. One patient, who remained dissatisfied due to an aesthetic mismatch, illustrates how a perceived failure in one dimension can taint the entire treatment experience, even when functional success is achieved. For such patients, even minor deviations in crown shade or gingival contouring can lead to disproportionate dissatisfaction. 

This study also confirms that aesthetic outcomes are a key concern for patients receiving implants in the anterior maxilla, often becoming the single most scrutinized element of care. Meanwhile, those treated in posterior regions prioritized function, particularly the ability to chew without discomfort or restriction. The importance of chewing efficiency and dietary freedom has been well documented in previous reports, such as those by Moraschini et al. [16]. Interestingly, patients who experienced aesthetic mismatches were less likely to forgive clinicians, whereas those facing logistical delays or functional adaptation challenges often rationalized their discomfort. This suggests that aesthetic dissatisfaction may be perceived as a more permanent and visible failure, while logistical and functional issues are considered transient and manageable. 

The role of clinician-patient communication emerged as a critical determinant of satisfaction. Participants who felt included in the decision-making process and adequately informed expressed a higher degree of contentment, consistent with earlier findings [19]. One participant highlighted the difficulty of interacting with clinicians due to language barriers, which undermined trust and hindered emotional expression. This is particularly relevant in multilingual healthcare environments such as India, where language mismatch may prevent patients from voicing concerns or seeking clarification [21]. While clinicians may view such interactions as routine, patients, especially those with limited literacy or language fluency, experience them as emotionally charged events that shape their perception of care quality. This supports the call for greater sensitivity training and communication aids (e.g., multilingual consent forms, interpreters) in tertiary care centres [21, 22]. 

Waiting times, appointment delays, and travel burden were common frustrations, particularly among patients from rural or distant regions. While most patients were ultimately forgiving, suggesting a high degree of resilience and trust in tertiary care, these inefficiencies remain problematic. They reduce perceived responsiveness and can alienate patients, especially when not adequately explained [23]. While not inherently clinical failures, such events heavily impact satisfaction, underscoring the need for holistic system-level interventions to improve coordination, scheduling, and patient support services. 

Despite isolated dissatisfactions, 27 of the 30 patients stated they would recommend implant therapy at the centre, a powerful testament to the overall perceived value of the treatment. This reinforces the concept that even when imperfections occur, patients’ final judgments hinge on whether they felt heard, helped, and respected [20,25]. A patient might have endured procedural delays or functional adaptation struggles but still enthusiastically endorse the hospital if they felt the care team was compassionate and competent. This highlights the value of post-treatment interviews as a feedback mechanism for quality improvement. 

Implications of the study findings

Training in Empathetic Communication

Clinicians should be sensitized to the emotional needs of implant patients and trained in culturally competent communication. Even minimal efforts, such as introducing oneself clearly, explaining procedures in simple language, or validating patient concerns, can significantly improve satisfaction. 

Improved Aesthetic Planning

Particularly for anterior implants, patients must be shown realistic outcomes via digital previews or mock-ups to calibrate expectations. Post-restoration dissatisfaction often stemmed not from clinical inaccuracy but from unmet visual expectations. 

Operational Reforms

Tertiary centres should streamline appointment systems, minimize cancellations, and offer assistance (like SMS reminders or travel coordination) to reduce logistical burden on patients. 

Integrating PROMs With Narrative Feedback

PROMs should be supplemented by qualitative follow-ups to capture subjective and emotional feedback. Such strategies yield a more comprehensive understanding of patient satisfaction [20, 24, 25]. 

Tailored Postoperative Instructions

Linguistically adapted and visually rich post-operative care guides can ensure better compliance, particularly among low-literacy or non-native patients. 

Limitations

This study is limited by the fact that it is a purely qualitative study and does not consider the quantitative metrics of implant placement, like implant survival rates. It is also noticed that the aesthetic demands of an anterior tooth are vastly different compared to a posterior tooth. Aesthetic concerns for a young adult, for instance, are different of someone in their 70s. Since this study does not have a mixed-method design, it fails to provide a comprehensive understanding of the entire treatment process of the patient, which should ideally be a holistic evaluation of the treatment protocol, combining both qualitative and quantitative aspects of research. Moreover, trustworthiness is supported by double coding, but the single-centre focus may restrict generalizability.

## Conclusions

Dental implant therapy in the upper and lower arches provides profound benefits not only in restoring oral function and aesthetics but also in improving patients' emotional and social well-being. While the clinical success rate remains high, true treatment success must incorporate the patient's satisfaction as well as a holistic perspective regarding the treatment as a whole - ranging from aesthetic harmony to communication clarity, appointment logistics, and emotional validation. Most patients emerged from the implant experience feeling transformed - socially, emotionally, and functionally. However, for a minority, gaps in communication, aesthetic misalignment, or systemic inefficiencies tainted otherwise successful outcomes. These findings suggest that implant dentistry should adopt a more patient-centric approach, integrated with a balanced patient-doctor rapport, so that patients continue with maintenance therapy post-implant placement. Findings are exploratory and hypothesis-generating within tertiary implant settings. By integrating emotional intelligence, improved logistics, aesthetic transparency, and cultural sensitivity into practice, clinicians can ensure that the patient experience matches the clinical excellence of implant therapy.

## Appendices

Interview questionnaire

The complete interview questionnaire was as follows:

Hello. I have come as an investigator for the study entitled “Patient Satisfaction with Dental Implants in the Upper and Lower Arches Placed in a Tertiary Care Setting: A Qualitative Study”. We will use this information only for the benefit of society. I also appreciate your consent to make an audio recording of the interview, to ensure that I do not lose any information.

Q1. How were you first introduced to the implant surgery treatment?

Q2. How was your entire treatment experience from your perspective? How was the behaviour of the staff in general? Were the costs of the procedure, including treatment methodology, mentioned clearly by the doctor?

Q3. How do you feel about the duration for waiting for the crown placement?

Q4. How did you find the colour of the crown to be with respect to the anterior region?

Q5. How did you find the difference between the natural teeth and the implant?

Q6. Do you have any food accumulation with the implant?

Q7. Do you find any difficulty in chewing or biting into food?

Q8. Do you find the implant teeth aesthetic?

Q9. Do you maintain your oral hygiene post the implant treatment in any new way? If yes, then in what way has your maintenance changed?

Q10. How often do you visit your dentist for check-ups?

Okay, with that, we come to the end of our survey. Thank you very much for spending your valuable time with us. Have a nice day.

SRQR reporting checklist for qualitative studies

Table 2 contains the SRQR checklist.

**Table 2:** The SRQR Reporting Checklist for Qualitative Studies SRQR: Standards for Reporting Qualitative Research

Parameter	Item Description	Location (or reason for not reporting)
Title and Abstract		
Title: Qualitative Study on Patient Satisfaction with Dental Implants	The title, "Patient Satisfaction with Dental Implants in the Upper and Lower Arches Placed in a Tertiary Care Setting: A Qualitative Study," clearly identifies the study as qualitative and specifies the focus on patient satisfaction with dental implants in maxillary and mandibular regions at a tertiary care hospital in Kerala, India.	Title Page
Abstract: Summary of Patient Satisfaction with Dental Implants	The abstract outlines the study’s background, noting dental implants’ high survival rates but emphasizing patient satisfaction’s multidimensional nature; describes the qualitative methodology using semi-structured interviews with 30 patients; reports key findings, including 26 positive experiences, themes of aesthetics, function, and logistics; and concludes that holistic evaluation improves care delivery.	Abstract
Introduction		
Problem Formulation: Patient Satisfaction in Dental Implant Therapy	Patient satisfaction with dental implants is a critical outcome beyond clinical success (90–98% survival rates), encompassing aesthetic, functional, emotional, and logistical dimensions. Prior research focuses on quantitative metrics like bone loss, but qualitative studies are limited, missing nuanced patient experiences, especially in tertiary care settings with diverse populations. Literature highlights discrepancies between clinical and patient aesthetic assessments, emotional impacts of tooth loss, and system-level barriers like delays, underscoring the need for this study.	Introduction, paragraphs 1–5
Purpose or research question: Exploring Lived Experiences of Dental Implant Patients	The study aims to explore the lived experiences of 30 patients who received dental implants in anterior and posterior maxillary/mandibular regions at a tertiary care hospital, focusing on their satisfaction across aesthetic (e.g., smile appearance), functional (e.g., chewing), emotional (e.g., confidence), and logistical (e.g., travel, delays) dimensions to inform patient-centered care.	Introduction, paragraph 6
Methods		
Qualitative approach and research paradigm: Interpretivist Paradigm for Dental Implant Study	The study employs thematic analysis within an interpretivist paradigm, which views patient satisfaction as subjective and context-dependent, to capture diverse experiences of dental implant patients. This approach was chosen to allow patients to articulate their narratives freely, aligning with the exploratory goal of understanding satisfaction in a tertiary care context.	Materials and Methods, Study Design
Researcher characteristics and reflexivity: Influence of Dental Researchers	The research team, comprising postgraduate residents and faculty in periodontics, has clinical expertise in implantology, which may shape interpretations toward functional outcomes. To mitigate bias, researchers maintained a neutral tone during interviews, used open-ended questions, and employed double coding and peer debriefing to ensure findings reflected patient perspectives, not clinical assumptions.	Materials and Methods, Data Collection
Context: Tertiary Care Hospital in Kerala for Dental Implant Study	The study was conducted at a private tertiary care hospital in Kerala, India, serving a diverse patient population with varied socioeconomic, linguistic (e.g., Malayalam, English), and geographic backgrounds, some traveling over 100 km. This setting was chosen to reflect real-world challenges like language barriers and logistical delays, which influence patient satisfaction with dental implants.	Materials and Methods, Study Design
Sampling strategy: Purposive Sampling of Dental Implant Patients	Thirty patients (17 females, 13 males, aged 35–80) were purposively selected based on having received at least one dental implant in anterior or posterior maxillary/mandibular regions, with prosthetic restoration completed at least 6 months prior, and willingness to participate. This ensured diverse experiences.	Materials and Methods, Participant Selection
Ethical issues pertaining to human subjects: Ethics in Dental Implant Research	All 30 participants provided written informed consent after receiving study information in English or Malayalam. Data were anonymized during transcription and analysis to protect confidentiality, with audio recordings stored securely.	Materials and Methods, Study Design
Data collection methods: Semi-Structured Interviews for Dental Implant Experiences	Semi-structured interviews, lasting 20–45 minutes, were conducted with 30 patients to explore their experiences with dental implants, focusing on treatment experience, aesthetics, function, logistics, communication, and recommendations. Conducted in English or Malayalam by trained researchers, the open-ended format allowed flexibility to capture diverse narratives, chosen to reflect patients’ priorities.	Materials and Methods, Data Collection
Data collection instruments and technologies: Interview Guide and Audio Recorders for Dental Implant Study	A flexible interview guide was developed to explore patient satisfaction with dental implants, covering topics like aesthetic outcomes, chewing ability, and hospital logistics. Audio recorders captured interviews in English or Malayalam, ensuring accurate data. The guide remained consistent throughout, with no modifications reported, to maintain comparability across interviews.	Materials and Methods, Data Collection
Units of study: Dental Implant Patients	The study included 30 patients (17 females, 13 males, aged 35–80) who received dental implants in anterior (19) or posterior (11) maxillary/mandibular regions, with restorations completed at least 6 months prior. All participants completed full interviews, reflecting diverse socioeconomic and linguistic backgrounds, some from rural areas over 100 km away.	Materials and Methods, Participant Selection; Results, Participant Characteristics
Data processing: Managing Dental Implant Interview Data	Interview audio was anonymized and transcribed verbatim in English or Malayalam. Transcripts were coded using QDA Miner Lite, with data managed securely to protect confidentiality. Two researchers independently coded transcripts, comparing results for consistency.	Materials and Methods, Thematic Analysis
Data analysis: Thematic Analysis of Dental Implant Experiences	Thematic analysis identified five themes from interview transcripts: overall treatment experience, treatment coordination, aesthetic outcomes, functional challenges, and recommendations. Two researchers independently coded data using QDA Miner Lite, reconciling discrepancies via discussion. This method was chosen to systematically organize diverse patient narratives into actionable insights.	Materials and Methods, Thematic Analysis
Techniques to enhance trustworthiness: Ensuring Credibility in Dental Implant Study	Double coding by two researchers ensured coding consistency, and peer debriefing among the research team validated themes. Member checking was not used due to logistical constraints. These methods were selected to enhance credibility by reducing individual bias and ensuring findings aligned with patient narratives.	Materials and Methods, Data Collection
Results		
Synthesis and interpretation: Themes of Dental Implant Patient Satisfaction	Five themes emerged: 1) Overall treatment experience (26 positive, 1 neutral, 3 negative); 2) Treatment coordination (delays, travel issues); 3) Aesthetic outcomes (19 satisfied, 7 with color mismatches); 4) Functional challenges (chewing, biting difficulties); 5) Recommendations (27 would recommend). Findings link to prior research on patient-centred outcomes, highlighting emotional and logistical impacts.	Results
Links to empirical data: Patient Quotes in Dental Implant Study	Patient quotes illustrate findings, e.g., Patient 14 (female, 48) on aesthetic success: “I smile confidently… it feels like a second chance”; Patient 8 (male, 55) on logistical issues: “I couldn’t ask questions properly—I just said yes to everything because I was confused.” These substantiate themes of satisfaction and challenges.	Results
Discussion		
Integration with prior work, implications, transferability, and contribution(s) to the field: Impact of Dental Implant Study	Findings align with prior studies showing high implant satisfaction (86.7% positive) but reveal nuances like aesthetic dissatisfaction’s emotional impact. Implications include improved communication, aesthetic planning, and logistics. Contributions emphasize patient-centered care in tertiary settings.	Discussion
Limitations: Limitations of Dental Implant Qualitative Study	The study’s qualitative design excludes quantitative metrics like implant survival rates, limiting holistic evaluation. Trustworthiness is supported by double coding, but the single-centre focus may restrict generalizability. Additional methods like mixed-methods could enhance comprehensiveness.	Discussion
Other		
Conflicts of interest: Conflicts in Dental Implant Study	No conflicts of interest were identified among the research team, ensuring unbiased conduct and reporting of the study on patient satisfaction with dental implants. All authors declared no financial or personal influences that could affect the study.	Conflicts of Interest
Funding: Funding for Dental Implant Study	The study received no external funding, relying on institutional resources only. No funders influenced study design, data collection, analysis, or reporting, ensuring independence of findings.	Funding
